# Palmitoylethanolamide causes dose-dependent changes in brain function and the lipidome

**DOI:** 10.3389/fnins.2024.1506352

**Published:** 2024-11-27

**Authors:** Shreyas Balaji, Taylor J. Woodward, Emily Richter, Arnold Chang, Richard Otiz, Praveen P. Kulkarni, Kaashyap Balaji, Heather B. Bradshaw, Craig F. Ferris

**Affiliations:** ^1^Center for Translational Neuroimaging, Northeastern University, Boston, MA, United States; ^2^Department of Psychological and Brain Sciences, Program in Neuroscience, Indiana University, Bloomington, IN, United States; ^3^Department of Psychology, Northern Illinois University, DeKalb, IL, United States; ^4^Departments of Psychology and Pharmaceutical Sciences, Northeastern University Boston, Boston, MA, United States

**Keywords:** cerebellum, functional connectivity, BOLD imaging, sensorimotor cortex, lipidomics, endocannabinoids

## Abstract

The present studies were undertaken to understand the effects of the commonly used nutraceutical PEA on brain function and lipid chemistry. These studies using MRI and broad-scale lipidomics are without precedent in animal or human research. During the MRI scanning session awake rats were given one of three doses of PEA (3, 10, or 30 mg/kg) or vehicle and imaged for changes in BOLD signal and functional connectivity. There was an inverse dose–response for negative BOLD suggesting a decrease in brain activity affecting the prefrontal ctx, sensorimotor cortices, basal ganglia and thalamus. However, there was a dose-dependent increase in functional connectivity in these same brain areas. Plasma and CNS levels of PEA and over 80 endogenous lipids (endolipids) were determined post treatment. While levels of PEA in the CNS were significantly higher after 30 mg/kg treatment, levels of the endocannabinoid, Anandamide, and at least 20 additional endolipids, were significantly lower across the CNS. Of the 78 endolipids that were detected in all CNS regions evaluated, 51 of them were modulated in at least one of the regions. Taken together, the functional connectivity and lipidomics changes provide evidence that PEA treatment drives substantial changes in CNS activity.

## Introduction

Palmitoylethanolamide (PEA) is an endogenous fatty acid amide, belonging to a class of lipid signaling molecules, the *N*-acylethanolamines (NAEs) that includes the endogenous cannabinoid anandamide (AEA) (Rahman et al., 2014). There are numerous preclinical studies showing PEA is an effective analgesic in various rodent models of pain (Jaggar et al., 1998; Conti et al., 2002; Ardizzone et al., 2021; Costa et al., 2008; D'Amico et al., 2020; Farquhar-Smith and Rice, 2001; Cristiano et al., 2022; Calignano et al., 1998). Well controlled, clinical trials report PEA can reduce chronic pain under various conditions (Scuteri et al., 2022; Pickering et al., 2022; Masek et al., 1974). PEA is also an effective anti-inflammatory agent (Conti et al., 2002; Ardizzone et al., 2021; Costa et al., 2002; Lo Verme et al., 2005) and has been used as an anticonvulsant in response to chemical and electroshock induced seizures in rodents (Lambert et al., 2001; Post et al., 2018; Sheerin et al., 2004).

The actual mechanism of action for PEA, i.e., molecular targets, and neural circuits, is not fully understood. PEA is an opportunistic molecule that impacts multiple signaling pathways (Bradshaw et al., 2013) Exogenously delivered PEA is hypothesized to affect biosynthetic and metabolic pathways on lipid pathways in part by enhancing the levels of AEA by competing with FAAH the primary enzyme for degrading both NAEs (Rankin and Fowler, 2020). Though this view leaves out many of the other enzymatic pathways open to these types of endolipids and how the modulation of one can influence others (Bradshaw and Leishman, 2017). While not considered a canonical endocannabinoid, PEA’s analgesic effects on rodents may act through a CB2-like receptor that has not been fully characterized (Farquhar-Smith et al., 2002). The anti-inflammatory effects of PEA are hypothesized to be mediated, at least in part, through nuclear receptor, peroxisome proliferator-activator receptor-α (PPARα) (Lo Verme et al., 2005), PPAR-α acts on macrophages to inhibit proinflammatory cytokines. Ultimately, the mechanisms of action of this ubiquitous endolipid are largely unknown and its use as an exogenous drug likely engages additional systems.

Functional magnetic resonance imaging in awake animals provides a mean of evaluating the effect of exogenous PEA on global brain activity (Ferris, 2022). Specifically, pharmacological MRI (phMRI) provides a view of the integrated neural circuits that respond in a dose-dependent manner to a test compound (Brems et al., 2023; Sathe et al., 2023; Kawazoe et al., 2022). When combined with resting state functional connectivity (rsFC) the key nodes and connections in these circuits can be identified. While phMRI and rsFC are agnostic with respect to the mechanism(s) of an opportunistic molecule like PEA the data can be used to make prediction about molecular and behavioral outcomes. To date there has been no functional imaging studies on animals to characterize the effect of exogenous treatment of PEA on the brain. To our knowledge, the only human MRI study was conducted by Alshelh and colleagues where PEA was given for 6 weeks to treat neuropathic pain (Alshelh et al., 2019). The slow oscillations in the pattern of thalamic bursting activity associated with neuropathy were altered by PEA together with the functional connections between the brainstem and the thalamus. These changes in functional brain activity were associated with a reduction in neuropathic pain.

The present studies were undertaken to characterize or “fingerprint” the functional activity of exogenous PEA on brain activity in awake rats using BOLD imaging and to investigate mechanisms of action through the evaluation of the CNS lipidome. These studies on the acute dose-dependent effects of PEA on brain function using MRI and its broadscale effects on the CNS lipidome are without precedent. Given the reported behavioral effects of exogenous PEA, we hypothesized it would interact with neural circuitry associated with pain, e.g., parabrachial n. periaqueductal gray, ventral posterolateral thalamus as identified in other awake imaging studies on rodents (Becerra et al., 2011; Chang et al., 2017; Yee et al., 2015; Alkislar et al., 2020), and the anterior thalamus and other key node associated with the genesis of clonic–tonic seizures (Ferris and Tenney, 2013; Brevard et al., 2006; Tenney et al., 2003). Exogenous PEA affected all of these sites, but to our surprise the response was an inverse dose dependent negative BOLD with the lowest dose (3 mg/kg) having the greatest effect, while the highest dose of PEA presented with the greatest functional connectivity. The acute behavioral effects on motor activity were most robust in the highest dose. The effects on the CNS lipidome were focused on the 30 mg/kg dose and illustrated that the overall levels of PEA were increased across the 6 areas of the CNS tested as well as a differential modulatory effect on over 50 endogenous lipids. Together, these data provide a unique insight into the effects of an endogenous lipid that drives significant effects in the molecular and physiological activity of the brain when given exogenously.

## Materials and methods

### Animals

Adult female Sprague Dawley rats (*n* = 68), 90–100 days of age were purchased from Charles River Laboratories (Wilmington, MA, United States). Rats were kept in Plexiglas cages, with two rats per cage, and maintained at a room temperature of 22–24°C under a 12:12 reverse light:dark cycle, with lights turning off at 09:00 h. All experimental activities were conducted between 10:00 and 18:00 h under dim red illumination to prevent disturbances due to transitions between light–dark cycles. The rats had access to food and water without restriction. All animal acquisition and care procedures adhered to the guidelines outlined in the NIH Guide for the Care and Use of Laboratory Animals. All the methods and procedures described in this study had been approved in advance by the Northeastern University Institutional Animal Care and Use Committee, with the protocol assigned the number 20-0626R. Northeastern University’s animal care and housing facilities have full accreditation from AAALAC, International. The study protocols employed in this research followed the ARRIVE guidelines for reporting *in vivo* experiments in animal research as recommended by Kilkenny et al. (2010). Animals were monitored daily over the duration of the study for general health, food and water consumption. A 15% loss in body weight was set as a humane endpoint.

### Palmitoylethanolamide preparation and administration

PEA was provided by RAD Wellness (Valleyview, TX). On the day of imaging PEA was taken up in a solution of 2% gum Arabic in water. To deliver drug remotely during the imaging session, a poly-ethylene tube (PE-20), approximately 30 cm in length, was positioned in the peritoneal cavity. There were four experimental groups: vehicle, 3.0 mg, 10 mg, and 30 mg/kg PEA. The range of doses of PEA were taken from the literature (Jaggar et al., 1998; Conti et al., 2002; Farquhar-Smith and Rice, 2001; Costa et al., 2002; Ahmad et al., 2012). Rats were randomly assigned to one of four experimental groups, six per group. Due to motion artifact five rats were excluded from the study: two from vehicle, two from 3.0 mg PEA and one from 30 mg PEA. The final subject distribution was: (1) gum Arabic vehicle, *n* = 4; (2) 3.0 mg PEA, *n* = 4; (3) 10 mg PEA, *n* = 6; and (4) 30 mg PEA, *n* = 5. The behavioral studies included 24 rats again randomly divided into four experimental groups of six each, while the lipidomics studies included 20 rats randomly divided into four groups of five in each. These rats were independent of those used for imaging.

### Acclimation for awake imaging

To mitigate the stress associated with head restraint, rats underwent an acclimation protocol to familiarize them with the restraining system, which consisted of a head holder and body tube. This system was designed to include a cushioned head support, eliminating the need for ear bars and, in turn, reducing discomfort to the animals while minimizing any unintended motion artifacts. These acclimation sessions were conducted daily for four to five consecutive days. During these sessions, rats were briefly anesthetized with 1–2% isoflurane and securely placed into the head holder, with their forepaws fastened using surgical tape.

Once fully conscious, the rats were positioned within an opaque black box, essentially a “mock scanner,” for 30 min. Inside the mock scanner, a tape recording of the MRI pulse sequence was played to simulate the environment of the magnet bore and the imaging protocol. It is worth noting that significant decreases in respiration, heart rate, motor activity, and plasma corticosterone levels were observed when comparing the first and last acclimation sessions, as reported by King et al. (2005). This reduction in autonomic and somatic indicators of arousal and stress contributed to the enhancement of signal resolution and image quality.

The effectiveness of this passive restraining system can be evaluated by the minimal levels of motion artifacts observed during the 20-min imaging sessions. Supplementary Figure S1 illustrates the average displacement in all orthogonal directions throughout the entire 20-min scanning period for all rats, whether they received PEA or a vehicle (*n* = 19). At no time point did the average motion exceed 200 μm in any orthogonal direction.

### Image acquisition

Five to six rats were imaged in a day. Each day had a mix of the different experimental groups known by all the investigators. A detailed description of the awake rat imaging system is published elsewhere (Ferris et al., 2014). Notably, we used a quadrature transmit/receive volume coil (ID = 38 mm) that provided both high anatomical resolution and high signal-to-noise ratio for voxel-based BOLD fMRI (Ekam Imaging Boston MA). The design of the coil provided complete coverage of the brain from olfactory bulbs to brain stem with excellent B1 field homogeneity. A video of the rat preparation for imaging is available at www.youtube.com/watch?v=JQX1wgOV3K4. The experiments were carried out with the use of a Bruker Biospec 7.0 T/20-cm USR horizontal magnet, accompanied by a 2 T/m magnetic field gradient insert with an inner diameter of 120 mm, capable of a rapid 120-μsec rise time. At the outset of each imaging session, a high-resolution anatomical dataset was obtained employing the rapid acquisition, relaxation enhancement (RARE) pulse sequence (RARE factor 8). This involved acquiring 25 slices, each with a thickness of 1 mm, within a field of view (FOV) of 3.0 cm^2^. The data matrix used was 256 × 256, with a repetition time (TR) of 3 s, an echo time (TE) of 12 milliseconds, and an effective TE of 48 milliseconds. The number of excitations (NEX) was set to 3, resulting in an acquisition time of approximately 4.48 min, and an in-plane resolution of 117.2 μm^2^.

Functional images were captured using a multi-slice Half Fourier Acquisition Single Shot Turbo Spin Echo (HASTE) pulse sequence. This involved collecting 22 slices, each 1.1 mm thick, with the same FOV of 3.0 cm^2^. The data matrix was 96 × 96, with a TR of 6 s, TE of 3.75 milliseconds, and an effective TE of 22.5 milliseconds. The acquisition took approximately 20 min, with an in-plane resolution of 312.5 μm^2^. It should also be emphasized that high neuroanatomical fidelity and spatial resolution are critical in identifying distributed neural circuits in any animal imaging study. Many brain areas in a segmented rat atlas have in-plane boundaries of less than 400 μm2 and may extend for over 1,000 μm in the rostral/caudal plane. With the development of a segmented, annotated 3D MRI atlas for rats (Ekam Solutions, Boston, MA, United States), it is now possible to localize functional imaging data to precise 3D “volumes of interest” in clearly delineated brain areas. For example, this spatial resolution was sufficient to identify the bilateral habenula of the thalamus, with approximately 4–5 voxels on each side, but not to differentiate between the lateral and medial habenula (Kawazoe et al., 2022). Using the spin-echo technique for functional imaging was essential to ensure high anatomical accuracy for data registration to the rat MRI atlas.

Each functional imaging session entailed continuous data acquisition for 250 scan repetitions, covering a total time of 25 min. The first 5 min of this period constituted a baseline, consisting of the initial 50 image acquisitions, followed by the administration of either a vehicle or a drug, and then another 200 acquisitions over the subsequent 20 min. The 20 min imaging protocol is standard for lipid soluble drugs that rapidly cross the blood brain barrier (Sadaka et al., 2021). The order of drug doses was randomized across the scanning sessions.

Approximately 5 min after the 20-min functional imaging scan the rats were imaged for connectivity. The functional connectivity images were collected over a 15-min period. These functional connectivity scans were acquired using a spin-echo triple-shot EPI sequence with specific imaging parameters: a matrix size of 96 × 96 × 20 (height × width × depth), TR/TE of 1,000/15 milliseconds, voxel size of 0.312 × 0.312 × 1.2 mm, slice thickness of 1.2 mm, 150 repetitions, and a total acquisition time of 15 min.

### Imaging data analysis

The fMRI data analysis consisted of three main steps: pre-processing, processing, and post-processing. All these steps were executed using SPM-12 (available at https://www.fil.ion.ucl.ac.uk/spm/). In the pre-processing stage, several operations were performed, including co-registration, motion correction, smoothing, and detrending. Co-registration was carried out with specific parameters: Quality set at 0.97, Smoothing at 0.6 mm, and Separation at 0.4 mm. Additionally, Gaussian smoothing was applied with a Full Width at Half Maximum (FWHM) of 0.8 mm.

The processing step involved aligning the data to a rat atlas, followed by segmentation and statistical analysis. To achieve registration and segmentation, all images were initially aligned and registered to a 3D Rat Brain Atlas^©^, which included 173 segmented and annotated brain regions. This alignment was performed using the GUI-based EVA software developed by Ekam Solutions (Boston, MA). The image registration process encompassed translation, rotation, and scaling adjustments, performed independently in all three dimensions. All spatial transformations applied were compiled into a matrix [Tj] for each subject. Each transformed anatomical pixel location was tagged with its corresponding brain area, resulting in fully segmented representations of individual subjects within the atlas.


$$P_{i}\leq \frac{i}{V}\frac{q}{c\left(V\right)}\;\text{.}$$

In the equation, *Pi* represents the *p*-value derived from the *t*-test conducted at the *i*-th pixel within the region of interest (ROI), comprising *V* pixels, with each pixel ranked according to its probability value. For our analysis, we set the false-positive filter value *q* at 0.2, and we fixed the predetermined constant c(V) at unity, following a conservative approach for assessing significance (as per Sathe et al., 2023). Pixels that achieved statistical significance retained their relative percentage change values, while all other pixel values were set to zero. Our analysis employed a 95% confidence level, two-tailed distributions, and assumed heteroscedastic variance for the *t*-tests.

To create composite maps displaying the percent changes in the Blood Oxygen Level Dependent (BOLD) signal for each experimental group, we mapped each composite pixel location (in terms of rows, columns, and slices) to a voxel within the j-th subject using the inverse transformation matrix [Tj]-1. A trilinear interpolation method was used to determine the contribution of subject-specific voxel values to the composite representation. The use of inverse matrices ensured that the entire composite volume was populated with subject inputs. The average of all contributions was assigned as the percent change in the BOLD signal at each voxel within the composite representation of the brain for the respective experimental group.

**Figure 1:**
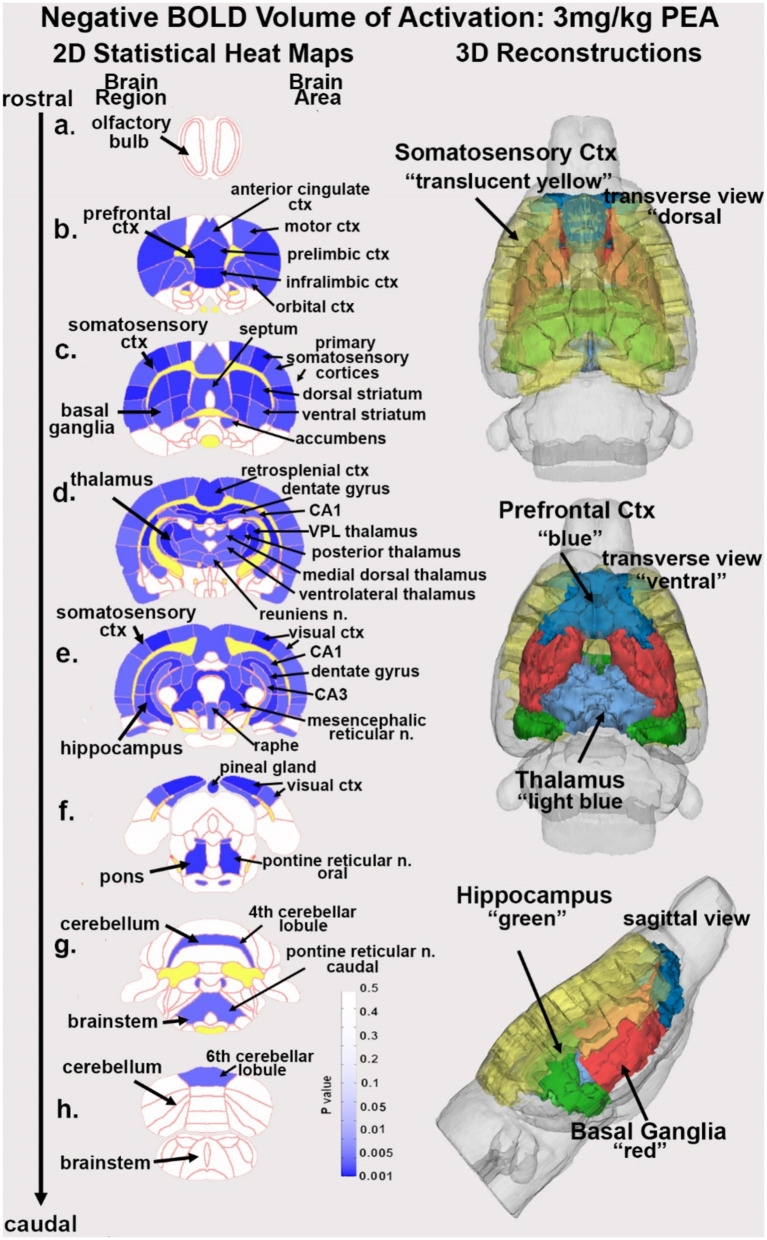
Change in BOLD signal with low dose PEA. Depicted are 2D heat maps of coronal sections (a-h) showing the location of brain areas that were significantly different in negative BOLD signal (blue highlight) between vehicle and low dose (3.0 mg/kg) PEA. These highlighted areas represent a PEA-induced increase in negative BOLD signal over vehicle. White matter tracts are highlighted in yellow. The brain regions are noted on the left of the section and brain areas on the right. The 3D color-coded reconstructions summarize the major brain areas that were significantly different. n. = nucleus.

In the post-processing phase, we compared the number of activated voxels in each of the 173 brain regions between the control and PEA doses using a Kruskal–Wallis test statistic. The data were ranked in order of significance, as detailed in Table 1. We generated probability heat maps, depicted in Figure 1, showing brain areas with significant differences when comparing two or more groups. The saturation of the pink color in these maps indicates a lower *p*-value, signifying higher confidence in the observed differences in those brain areas.

**Table 1:** Negative volume in BOLD signal.

	Vehicle		3 mg/kg		10 mg/kg		30 mg/kg			
Brain area	Ave	SE	Ave	SE	Ave	SE	Ave	SE	P val	Ω Sq
Posterior thalamus	2	1.5	74	13.4	85	5.3	43	13.2	0.005	0.515
CA2	1	0.5	4	0.6	6	0.7	3	0.7	0.006	0.664
Ventral medial striatum	1	0.3	97	6.0	70	15.0	52	17.0	0.006	0.664
Red n.	0	0.0	7	1.8	9	0.9	4	1.3	0.006	0.632
Parafascicular thalamus	1	0.5	40	9.9	50	8.2	25	8.6	0.007	0.572
Accumbens core	7	3.0	78	7.9	62	13.3	32	9.3	0.007	0.590
Retrosplenial rostral ctx	6	6.0	79	7.3	60	6.9	41	13.1	0.007	0.609
Reticular n. midbrain	18	6.0	161	38.4	150	11.2	74	27.1	0.008	0.456
Dorsal medial striatum	4	2.2	160	10.1	125	21.6	93	33.3	0.010	0.529
Dorsal lateral striatum	1	0.6	220	25.1	196	28.6	135	41.4	0.012	0.583
Dentate gyrus ventral	13	6.2	53	7.1	54	8.9	24	11.1	0.013	0.521
Claustrum	3	1.1	21	2.9	20	4.3	11	4.0	0.013	0.572
Prelimbic ctx	1	0.3	95	13.1	71	21.9	51	19.0	0.013	0.530
Ventral posteromedial thalamus	0	0.0	41	9.7	39	7.8	25	7.1	0.013	0.418
Anterior pretectal n.	1	1.3	19	2.3	22	1.9	12	5.1	0.014	0.565
Lateral posterior thalamus	3	3.0	52	11.2	53	8.1	30	13.6	0.014	0.451
Dentate gyrus dorsal	5	3.3	82	25.1	74	13.6	51	14.3	0.014	0.387
Superior colliculus	27	11.7	146	32.8	143	12.0	103	19.4	0.015	0.407
Primary motor ctx	45	27.1	333	44.0	259	55.6	198	58.2	0.015	0.470
Somatosensory ctx jaw	16	7.5	173	35.4	139	33.4	98	26.7	0.015	0.427
Somatosensory ctx hindlimb	6	6.0	81	12.3	65	9.7	64	13.2	0.016	0.524
Ventral posteriolateral thalamus	1	0.5	42	6.2	38	7.1	28	6.7	0.016	0.408
Pontine reticular n. oral	7	2.5	54	16.3	66	7.8	31	12.2	0.017	0.437
Somatosensory ctx upper lip	17	5.2	155	38.2	134	18.9	116	23.8	0.017	0.394
Ventrolateral thalamus	1	0.5	35	8.0	40	4.3	26	9.2	0.018	0.533
Zona incerta	0	0.0	32	11.5	18	9.2	12	4.3	0.018	0.359
CA1 dorsal	4	2.2	137	27.0	136	12.9	96	28.3	0.019	0.414
Visual 2 ctx	15	2.9	181	24.9	188	7.5	146	28.6	0.019	0.436
White matter, anterior	110	16.4	526	97.8	501	66.5	413	57.6	0.019	0.349
Retrosplenial caudal ctx	13	4.5	220	24.9	187	22.5	170	41.4	0.021	0.432
Somatosensory ctx barrel field	7	3.2	223	55.4	210	28.1	174	47.2	0.021	0.428
Parietal ctx	3	2.5	66	13.0	69	12.7	56	15.6	0.022	0.531
Temporal ctx	24	9.7	55	2.8	46	3.0	43	4.7	0.023	0.418
Ventral orbital ctx	16	6.2	63	5.2	61	8.3	29	12.8	0.023	0.392
Bed n. stria terminalis	0	0.0	31	3.4	28	11.7	22	10.7	0.024	0.469
Somatosensory ctx forelimb	12	11.2	135	24.9	113	19.1	90	26.6	0.024	0.433
Ventral lateral striatum	19	5.9	212	37.6	197	35.5	163	37.2	0.024	0.419
Visual 1 ctx	4	2.1	153	24.2	163	19.6	134	30.8	0.024	0.369
Somatosensory ctx trunk	0	0.3	12	3.2	13	3.8	10	2.3	0.025	0.483
Reticular n.	0	0.3	31	9.4	32	5.2	26	6.8	0.026	0.374
4th cerebellar lobule	54	4.6	95	13.7	97	8.1	53	21.2	0.026	0.355
White matter, posterior	8	3.9	69	23.6	74	8.0	51	18.4	0.026	0.390
CA3 hippocampus ventral	7	4.8	33	6.9	33	7.1	15	6.9	0.026	0.426
Globus pallidus	0	0.0	18	2.3	12	6.2	12	3.1	0.027	0.470
Lateral septal n.	6	5.8	115	27.9	93	25.9	82	32.8	0.028	0.391
Anterior thalamic nuclei	4	2.8	39	7.8	36	4.2	27	10.0	0.030	0.446
Extended amygdala	0	0.0	10	3.1	5	2.0	4	1.4	0.030	0.395
Secondary somatosensory ctx	12	6.6	97	26.6	111	10.7	94	13.2	0.030	0.322
Central amygdaloid n.	6	3.8	40	9.1	41	4.3	18	10.5	0.031	0.399
Ventral anterior thalamus	1	0.5	16	3.3	15	1.4	9	3.9	0.032	0.461
Infralimbic ctx	3	1.7	43	11.2	32	4.3	25	10.0	0.032	0.346
Medial dorsal thalamus	1	1.0	16	4.3	20	4.4	13	5.8	0.035	0.452
CA1 hippocampus ventral	26	12.8	94	5.1	74	12.9	44	21.3	0.035	0.357
Lateral geniculate	0	0.3	20	11.1	14	3.2	15	4.1	0.036	0.288
Subiculum dorsal	2	1.3	33	8.1	33	5.2	27	8.7	0.038	0.303
Raphe linear	0	0.0	9	3.5	8	1.5	4	2.0	0.038	0.372
Lateral orbital ctx	20	8.4	122	20.0	101	23.2	50	25.2	0.039	0.360
Insular ctx	123	10.1	295	81.5	317	44.8	247	34.8	0.039	0.362
Perirhinal ctx	43	18.9	98	17.1	103	8.3	57	20.2	0.040	0.329
Somatosensory ctx shoulder	2	2.3	18	1.2	13	3.5	14	3.2	0.040	0.434
CA3 dorsal	10	5.9	70	22.9	68	10.8	57	17.1	0.042	0.310
Anterior cingulate area	20	15.5	167	36.0	151	32.9	136	31.7	0.043	0.344
Locus coeruleus	0	0.3	0	0.0	2	0.6	1	0.4	0.043	0.417
6th cerebellar lobule	74	13.4	91	28.5	146	8.2	88	32.2	0.044	0.354
Auditory ctx	52	20.5	196	57.9	232	11.9	172	31.8	0.045	0.322
Reuniens n.	0	0.0	14	5.2	15	6.9	11	4.1	0.045	0.394
Ventromedial thalamus	0	0.0	28	7.9	25	7.1	16	5.8	0.046	0.330
Pontine reticular n. caudal	29	11.5	81	30.0	109	11.8	50	19.9	0.047	0.239
Triangular septal n.	0	0.0	5	1.9	3	1.1	4	1.5	0.047	0.412
Caudal piriform ctx	61	8.1	135	25.5	134	9.7	95	21.6	0.050	0.302

The dose-dependent effect of PEA on brain activity was quantified by assessing positive and negative percent changes in the BOLD signal relative to the baseline. Initial analyses of signal changes in individual subjects compared image acquisitions 150–200 to the baseline period of 5–45. The statistical significance of these changes was evaluated for each voxel, approximately 15,000 per rat in their original reference system, using independent Student *t*-tests. A threshold of 1% was applied to account for normal fluctuations in the BOLD signal in the awake rodent brain. To mitigate false positive detections resulting from multiple *t*-tests, a control mechanism was introduced to maintain the average false positive detection rate below 0.05, as defined by a specific formula.

### Resting state functional connectivity

#### Image acquisition

We collected scans using a spin-echo triple-shot EPI sequence, with the following imaging parameters: a matrix size of 96 × 96 × 20 (height × width × depth), a repetition time (TR) to echo time (TE) ratio of 1,000/15 milliseconds, a voxel size of 0.312 × 0.312 × 1.2 mm, a slice thickness of 1.2 mm, and a total of 200 repetitions, with a data acquisition time of 15 min.

For preprocessing, we utilized a combination of various software tools, including Analysis of Functional NeuroImages (AFNI_17.1.12), the FMRIB Software Library (FSL, v5.0.9), Deformable Registration via Attribute Matching and Mutual-Saliency Weighting (DRAMMS 1.4.1), and MATLAB. Brain tissue masks for resting-state functional images were manually delineated using 3DSlicer and applied for skull-stripping. We identified motion outliers, which are data segments affected by substantial motion, and recorded the corresponding time points for later regression. Large motion spikes were also detected and removed from the time-course signals. Following this step, slice timing correction was applied to account for interleaved slice acquisition order. We performed head motion correction using the six motion parameters, with the first volume serving as the reference image. Normalization involved registering functional data to the 3D MRI Rat Brain Atlas^©^ using affine registration through DRAMMS. After quality control, a band-pass filter (0.01 Hz to 0.1 Hz) was applied to reduce low-frequency drift effects and high-frequency physiological noise for each subject. The resulting images underwent detrending and spatial smoothing, with a full width at half maximum of 0.8 mm. Additionally, regressors, including motion outliers, the six motion parameters, the mean white matter, and cerebrospinal fluid time series, were incorporated into general linear models for nuisance regression to eliminate unwanted effects.

The region-to-region functional connectivity analysis was conducted to measure the correlations in spontaneous BOLD fluctuations. In this analysis, a network consists of nodes (brain regions of interest or ROIs) and edges (connections between regions). We averaged the voxel time series data within each node based on the residual images obtained through the nuisance regression procedure. Pearson’s correlation coefficients were computed across all pairs of nodes (14,535 pairs) for each subject within all three groups to assess interregional temporal correlations. The resulting *r*-values, ranging from −1 to 1, were z-transformed using Fisher’s Z transform to improve their normality. We constructed 166 × 166 symmetric connectivity matrices, with each entry representing the strength of an edge. Group-level analysis was then conducted to examine functional connectivity in the experimental groups. The Z-score matrices obtained from one-group t-tests were clustered using the K-nearest neighbors clustering method to identify how nodes cluster together and form resting-state networks. A Z-score threshold of |*Z*| = 2.3 was applied to eliminate spurious or weak node connections for visualization purposes.

### Functional connectivity analysis

#### Degree centrality

We conducted all network analysis using Gephi, which is an open-source software for network analysis and visualization. We imported the absolute values of the symmetric connectivity matrices for PEA and vehicle data, treating the edges as undirected networks. Degree centrality analysis measures the number of connections that a particular node has within the entire network. Degree centrality is defined as:


$$C_{D}\left(j\right)=\overset{n}{\underset{j=1}{\sum }}A_{ij}$$

Here, “*n*” represents the total number of rows in the adjacency matrix denoted as “*A*,” and the individual elements of the matrix are indicated as “*A_ij_*,” which signifies the count of edges connecting nodes *i* and *j*.

### Statistics

We conducted all statistical analysis for the graph theory assessment using GraphPad Prism version 9.1.2 for Windows (GraphPad Software, San Diego, California United States). To decide whether parametric or non-parametric assumptions were appropriate for different group subregions, we performed normality tests. We used Shapiro–Wilk’s tests to assess the normality assumption. Subregion degree centrality *p*-values exceeding 0.05 were considered to exhibit a normal distribution. Once the normality assumptions were confirmed, we employed paired t-tests to compare the degree centrality between the PEA and vehicle groups in various subregions. In cases where there was evidence against the normality assumption, we conducted a non-parametric Wilcoxon signed-rank (WSR) test.

### Behavioral studies

#### Open field test

The testing room for all behavioral studies was illuminated by red lights. The lighting Open Field testing was used to assess anxiety, exploratory behaviors, and locomotor ability (Seibenhener and Wooten, 2015). It is based on the natural tendency of an animal to explore and protect itself using avoidance which translates to a normal animal spending more time in the periphery of the open field along the walls of the arena than in the center (the most anxiogenic area). OF was conducted with 1 h after PEA treatment. Animals were placed in a large black cube-shaped Plexiglas box with no lid that was indirectly dimly illuminated with two 40 W incandescent red-light bulbs and allowed to explore for 20 min. For analysis, the arena was divided into a peripheral zone measuring 8 cm from the edge of the arena walls, and a central zone around 40% of the total surface of the arena. The amount of time spent in the periphery and the total distance were determined using ANY-MAZE tracking software. Each measure for the four experimental groups was compared with a one-way ANOVA using GraphPad Prism.

#### Novel object recognition test

The rats were first acclimatized to the enclosure for one day. In the familiarization phase, two identical objects were placed in opposite corners of the enclosure for a 5 min period where the rats were allowed to explore the object. The novel phase consisted of removing one familiar object and replacing it with a novel object. A higher exploration time of the novel object, measured by the rodent inhabiting the same corner of the object, was understood as a greater ability to discriminate between the novel and familiar object, indicating better memory performance. The box and objects were cleaned with 70% isopropyl alcohol between each rat exposure to eliminate olfactory cues. At the start of the study, each rat was placed in an empty 1764 cm^2^ Plexiglas box for 3 min each for habituation. There were two phases that each rat had to go through after habituation: the Familiar Phase and the Novel Phase. Twenty-four hours after habituation, the rats were placed in the same box with two identical objects for 5 min for familiarization. Two objects with different size, color, and texture were used for the NOP test. Half of each testing group was familiarized with an identical set of objects. Eighteen hours later, for the Novel Phase, the rats spent another 5 min in the box with one familiar object and one novel object. The Novel Phase was filmed and uploaded to ANY-Maze software for tracking and analysis. Recorded measures included total time spent investigating the novel object, total time investigating familiar object, number of investigations of each object, and discrimination between objects.

Investigation ratios (IR = time spent investigating the novel object/ time spent investigating both objects) were assessed using single-sample, two-tailed t-tests, and performance was compared to chance (i.e., IR = 0.5). An investigation ratio significantly greater than 0.5 indicates that the rats were spending more time with the novel object. Conversely, a ratio significantly smaller than chance was used as an index of a preference for the familiar object. Analysis was performed with GraphPad Prism.

### Tail flick

Tail flick was used to assess peripheral nociception. Rats were acclimated to a restrainer that restricts movement while also exposing the tail. The tail was placed on a hot plate set to 50°C. The time it took for the rat to flick its tail was recorded, with a ceiling time of 10 s. Tail flick was conducted within the first hour. of PEA treatment. Each measure for the four experimental groups was compared with a one-way ANOVA using GraphPad Prism.

### Lipid extraction and partial purification of plasma and CNS areas

Plasma and whole brains were shipped on dry ice to the Bradshaw lab where they were stored at −80°C until processed. Brains were thawed for 5 min on an ice-cold dissection plate, dissected into 9 brain regions, individual brain areas flash frozen in liquid nitrogen, then stored at −80°C as previously described. Plasma samples (75 μL) and brain areas [hypothalamus (HYP); cerebellum (CER), thalamus (THAL), right side anterior cortex (CTX), hippocampus (HIPP), and striatum (STR)] were processed as previously described (Bradshaw and Johnson, 2023; Leishman et al., 2019). In brief, methanolic extracts were partially purified using C18 solid phase extraction columns (SPEs; Agilent, Santa Clara, CA, United States). Final elutions (i.e., fractions) of 65, 75, and 100 percent methanol were collected and stored at −80°C until MS analysis.

### HPLC/MS/MS lipidomics analysis

Methanolic elutions were analyzed as previously described (Leishman et al., 2019) with the exception that the API 7500 (Sciex, Framingham, MA 01701, United States) was used for analysis instead of the API 3000. The API 7500 is coupled to a Shimadzu LC system LC-40DX3 (Kyoto, Japan). Examples of chromatograms of PEA standards as well as plasma and CNS samples from this instrumentation are illustrated in Supplementary Figures S2, S3. All parent and fragment pairs are identical to those previously described from the Bradshaw lab (Tortoriello et al., 2013). Standard curves were generated by using purchased standards (Cayman Chemical, Ann Arbor, MI, United States), and those made in-house were validated through NMR and MS analysis as previously described (Tan et al., 2006). Sicex Analyst peak matching software, Analyst (Sciex, Framingham, MA 01701, United States) was used to validate standard peaks and sample peaks.

### Statistical analysis for lipidomics

Statistical analyses for the plasma PEA dose curve were completed in IBM SPSS Statistics 29 (Chicago, IL, United States). One-way ANOVAs followed by Fisher’s Least Significant Difference post-hoc analyses were used to determine statistical differences between the average concentration of PEA measured in the plasma samples. Analysis of individual endogenous lipids in plasma and individual brain areas comparing the levels of a specific lipid in vehicle or after 30 mg/kg PEA injection were analyzed using Students t-tests set to 2-tails and Type 2. For the CNS combined analyses, levels of an individual lipid measured in the six different brain regions were summed to generate an overall value for each subject, therefore, the number of subjects remained the same at *n* = 5 each and analyzed via Students t-tests set to 2-tails and Type 2.

Samples with an endogenous lipid concentration outside of 2 standard deviations from the group mean were omitted from statistics for that compound. Statistical significance for all tests was set at *p* < 0.05, and trending significance at 0.05 < *p* < 0.10. Descriptive and inferential statistics were used to create heatmaps for visualizing changes in the concentration of each lipid analyte for every condition as previously described (Leishman et al., 2016). Briefly, the direction of changes for each analysis group compared to are depicted by color, with green representing an increase and orange representing a decrease. Level of significance is shown by color shade, wherein *p* < 0.05 is a dark shade and 0.05 < *p* < 0.1 is a light shade. Direction of the change compared to vehicle is represented by up (increase) or down (decrease) arrows. Effect size is represented by the number of arrows, where 1 arrow corresponds to 1–1.49-fold difference, 2 arrows to a 1.5–1.99-fold difference, 3 arrows to a 2–2.99-fold difference, 4 arrows a 3–9.99-fold difference, and 5 arrows a difference of tenfold or more (Stuart et al., 2013). Supplementary Figure S4 illustrates how these analyses are applied to the heatmaps. An abbreviation of ‘BDL’ indicates that the lipid concentration that was present in the sample was below the detectable levels of our equipment while ‘BAL’ indicates below analytical levels. Finally, bar graphs which show data as mean ± SE mean were made using GraphPad Prism Software (La Jolla, CA, United States) for key lipid families.

## Results

### Behavioral outcomes with acute PEA

**Figure 2:**
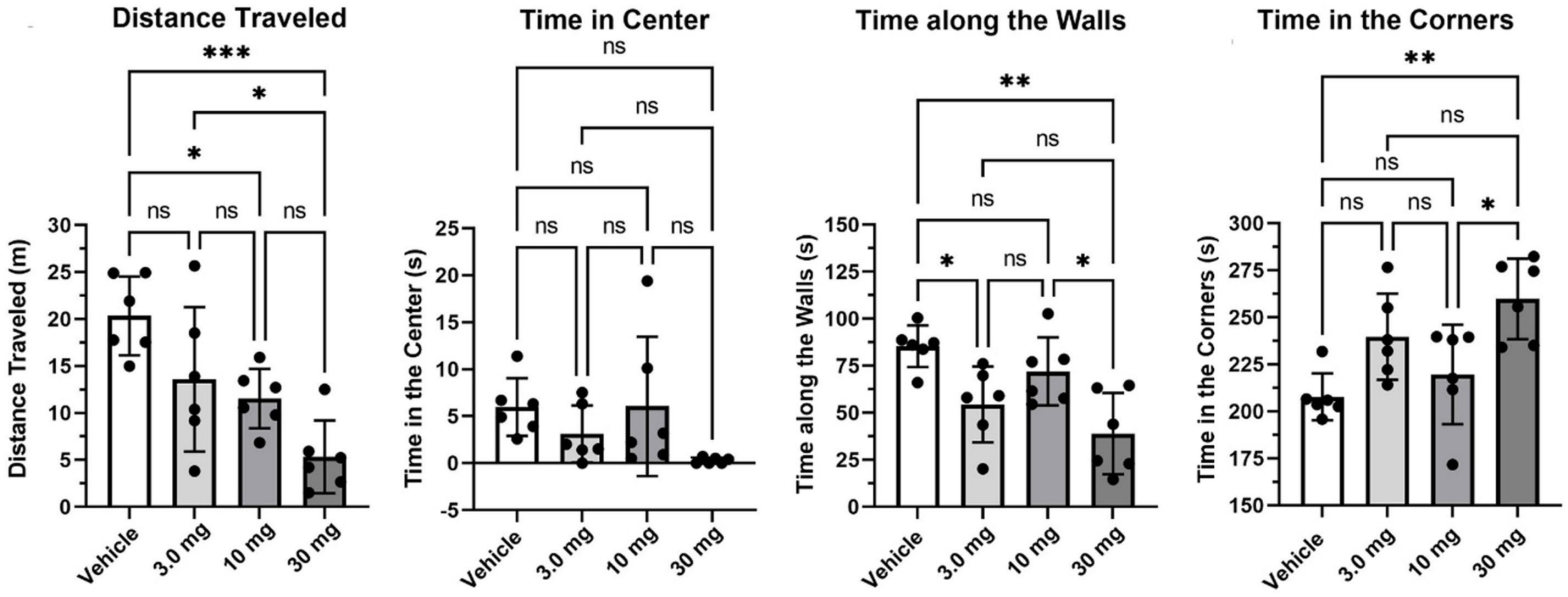
Open field. Shown are scatter plots (mean ± SD) of the dose-dependent changes in different measures of behavior recorded in the open field over a 5 min observation period. The black dots are each rat in the experiment (*p < 0.05; **p < 0.01), nonsignificant (ns). ***p < 0.001.

Figure 2 presents the dose-dependent variations (mean ± SD) in various behavioral measures recorded in the open field during a 5-min observation period. There was a significant dose-dependent decrease in distance traveled [*F*_(3, 20)_ = 9.02, *p* = 0.0006]. The highest 30 mg/kg dose of PEA was significantly less than vehicle (*p* < 0.0001), 3.0 mg/kg and 10 mg/kg (*p* < 0.05) treatments. Time spent along the walls was significantly different across treatments [*F*_(3, 20)_ = 7.47, *p* = 0.0015]. The 3 mg/kg dose was significantly less than vehicle (*p* < 0.05), while the high dose of 30 mg/kg showed the lowest mean time of 39 s and was significantly less than vehicle (*p* < 0.01) and the 10 mg/kg dose (*p* < 0.05). Time spent in the corner was also significantly different across treatments [*F*_(3, 20)_ = 2.46, *p* = 0.0918]. In this case, the high 30 mg/kg dose spent 260 s out of the total 600 s observation period standing in the corners of the open field box, a duration greater than vehicle (*p* < 0.01) and the 10 mg/kg dose (*p* < 0.05). There were no differences in time spent in the center (*F* = 2.466, *p* < 0.0918). Indeed, all rats, with the exception of two, spent less than 10/600 s exploring or crossing the center of the open field.

**Figure 3:**
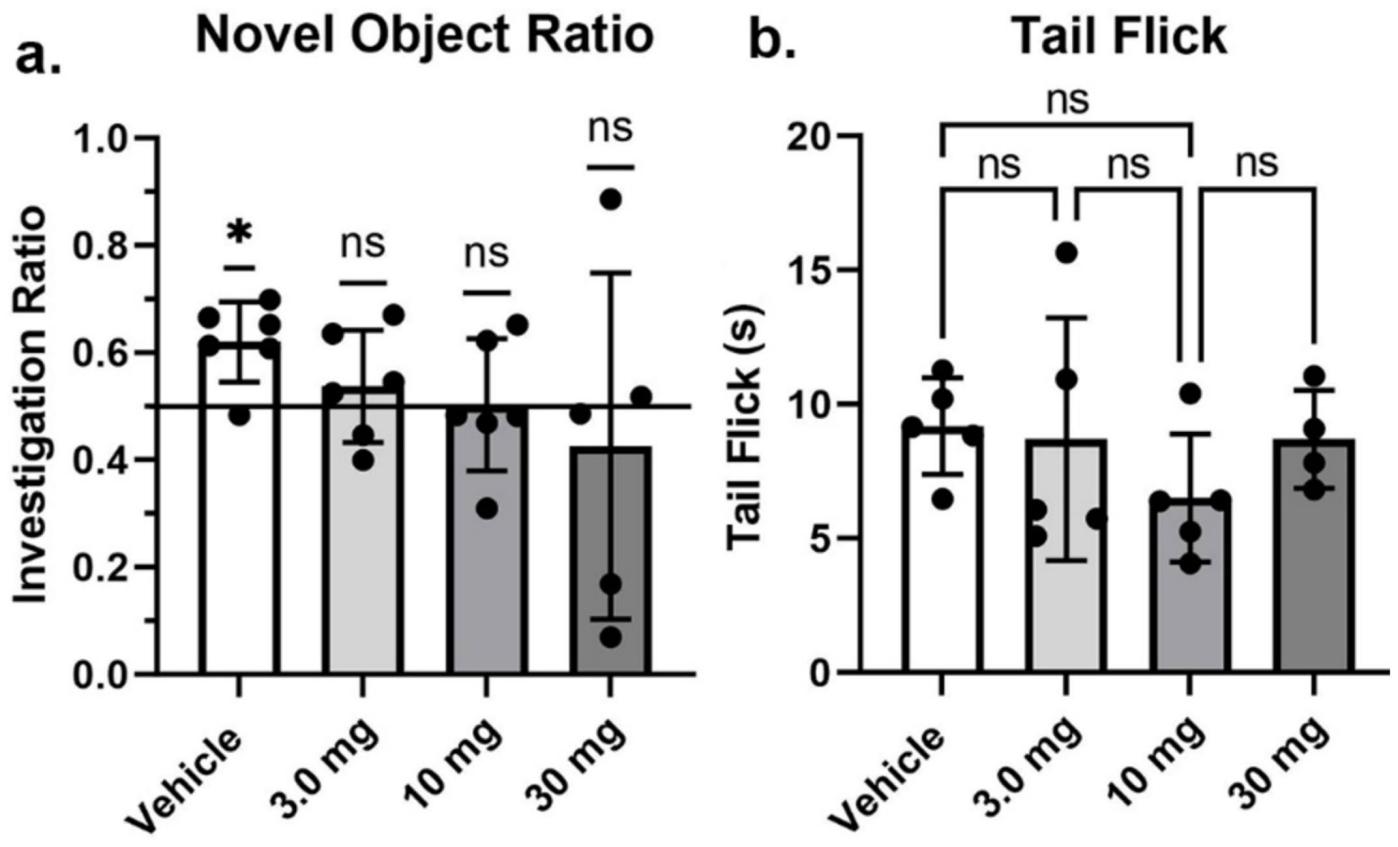
Novel object recognition and tail flick. Shown are scatter plots (mean ± SD) of the dose-dependent changes in the investigation ratio in the Novel Object Recognition task, and latency in seconds to withdraw the tail in the Tail flick assay. The black dots are each rat in the experiment (*p < 0.05), nonsignificant (ns).

Shown in Figure 3 are results (mean ± SD) from the Novel Object Recognition test assessing memory (Figure 3A), and tail flick (Figure 3B) to the stimulus of hot water assessing pain sensitivity. The investigation ratio or time spent in the vicinity of the novel object over the 5 min observation period was only greater than chance (0.50 horizontal line) in vehicle treated rats (*p* < 0.05) as determined by a one-sample two-tailed *t* test. Rats treated with the different doses of PEA failed to perform better than chance. Indeed, one of the rats in the high dose 30 mg/kg group had a zero investigation ratio as it failed to attend to the novel object and just sat in a corner for the 600 s observation period. This rat was omitted from the group analysis. With respect to pain sensitivity, there were no significant differences between treatments. All rats, with the exception of one, withdrew their tail under 15 s.

### Neuroimaging outcomes with acute PEA

Table 1 is a list of brain areas (Mean ± SE) that showed a dose-dependent change in negative volume of activation, i.e., number of negative BOLD voxels. The brain areas are ranked in order of their significance using a critical value of *p* < 0.05. Shown are their *p*-value and effect size given as omega square (Ω Sq). The false discovery rate (FDR) was *p* = 0.079. There were 69/173 brain areas shown to be significantly different with a Kruskal Wallace multiple comparisons test. For all PEA doses there is an increase in negative volume of activation over vehicle. When comparing the areas (mean values), note that many show an inverse dose–response, i.e., the 3 mg/kg dose has the highest voxel numbers and the 30 mg/kg dose the lowest. See for example, the ventral medial striatum, accumbens core and retrosplenial ctx. Certain brain regions like the thalamus, somatosensory cortices, hippocampus and basal ganglia containing the striatal areas and accumbens, are very sensitive to PEA (Figure 1).

Table 2 lists brain areas that showed a significant change in positive volume of activation, i.e., number of positive BOLD voxels in response to different doses of PEA. In this case there were only 26/173 brain areas that showed a *decrease* in positive volume of activation (FDR; *p* = 0.030). In all PEA doses the positive voxels were less than vehicle with the exception of the entorhinal ctx. The response to PEA was primarily blunting of the positive BOLD signal as many areas showed zero to few numbers of activated voxels such as the somatosensory ctx, anterior thalamus, and prelimbic ctx. The decrease in positive BOLD and increase in negative BOLD (Table 1) would suggest PEA is reducing activity in these brain areas. Tables for all 173 brain areas, negative and positive volume of activation, are provided in Supplementary material.

**Table 2:** Positive volume in BOLD signal.

	Vehicle		3 mg/kg		10 mg/kg		30 mg/kg			
Brain area	Ave	SE	Ave	SE	Ave	SE	Ave	SE	P val	Ω Sq
Parietal ctx	7	3.6	0	0.0	0	0.0	0	0.0	0.001	0.980
CA2	1	0.3	0	0.0	0	0.0	0	0.0	0.005	0.631
Retrosplenial rostral ctx	22	5.5	0	0.0	1	0.6	0	0.4	0.006	0.625
Triangular septal n.	3	1.3	0	0.0	0	0.0	0	0.0	0.006	0.624
Prelimbic ctx	14	3.9	0	0.0	1	1.6	2	1.7	0.009	0.561
Dentate gyrus ventral	8	2.9	5	3.4	1	0.7	16	5.8	0.009	0.555
CA3 hippocampus ventral	7	3.1	3	2.9	0	0.2	9	3.8	0.013	0.508
Somatosensory ctx hindlimb	9	4.8	0	0.3	0	0.0	0	0.0	0.017	0.469
Anterior thalamic nuclei	7	3.5	0	0.0	0	0.0	0	0.2	0.018	0.456
Secondary motor ctx	29	11.7	6	6.0	1	1.0	1	0.7	0.019	0.451
Reuniens n.	11	6.9	1	1.0	0	0.0	0	0.0	0.019	0.448
Anterior olfactory n.	23	5.1	10	4.8	3	1.8	16	5.3	0.021	0.434
Insular ctx	75	14.9	19	5.5	16	11.2	33	7.2	0.022	0.428
Accumbens core	6	3.3	0	0.3	0	0.0	4	2.3	0.023	0.422
Entorhinal ctx	90	33.2	20	6.6	57	8.7	108	29.8	0.025	0.411
Retrosplenial caudal ctx	108	29.1	8	4.5	11	6.9	13	6.9	0.025	0.410
Bed n. stria terminalis	6	5.0	1	0.5	0	0.0	0	0.0	0.026	0.407
Subthalamus	1	0.8	0	0.0	0	0.0	1	0.4	0.029	0.390
Lateral septal n.	49	11.9	14	10.7	7	3.0	9	2.3	0.031	0.381
Anterior cingulate area	29	11.1	2	1.4	4	2.1	0	0.3	0.038	0.349
Primary motor ctx	32	13.8	6	5.5	2	2.2	2	1.1	0.039	0.347
Lateral orbital ctx	9	2.9	2	1.8	1	0.6	5	3.2	0.044	0.327
White matter	27	11.6	8	3.6	5	2.0	15	4.0	0.045	0.325
Medial septum	3	1.7	0	0.0	0	0.0	0	0.0	0.047	0.318
Globus pallidus	5	4.4	0	0.0	0	0.0	0	0.0	0.048	0.316
Somatosensory ctx trunk	2	1.4	0	0.0	0	0.0	0	0.0	0.048	0.316

Figure 1 shows the anatomical location of the brain areas listed in Table 1 for negative BOLD volume of activation presented as 2D statistical heat maps. The coronal sections are labeled (a) through (h) and arranged from rostral (top) to caudal (bottom). Areas in blue are significantly different between VEH and 3 mg/kg dose of PEA. Areas in yellow denote the location of white matter tracts. The annotation is organized to report brain regions on the left and specific brain areas on the right. Brain section (a) shows no activity in the olfactory bulb. Section (b) highlights the prefrontal cortex comprised of the anterior cingulate, prelimbic, infralimbic and orbital cortices. Sections (c-e) highlight the many areas of the primary somatosensory ctx. Section (c) highlights the basal ganglia comprised of all striatal zones, i.e., dorsal medial, dorsal lateral, ventral medial, and ventral lateral together with the accumbens. Section (d) highlights the thalamus noting the ventral posterior lateral (VPL), posterior, medial dorsal and ventrolateral thalamic area all of which project to the primary somatosensory cortices. Sections (d-e) highlight the hippocampus noted by CA1, CA3, and dentate gyrus. Sections (e, f) denote the visual cortex. Note that the hindbrain sections (f-h) comprising the pons, cerebellum and hindbrain are not significantly affected by PEA. The essential findings taken from the 2D images, sections (b-e) are summarized in color-coded 3D reconstructions to the right. A dorsal view of the brain shows the somatosensory, motor and visual cortices as a yellow translucent cover over all of the brain minus the olfactory bulbs and cerebellum/brainstem. When this transverse perspective is viewed from below (ventral) or from the side (sagittal view) the prefrontal cortex (blue) basal ganglia (red) thalamus (light blue) and hippocampus (green can be seen).

**Figure 4:**
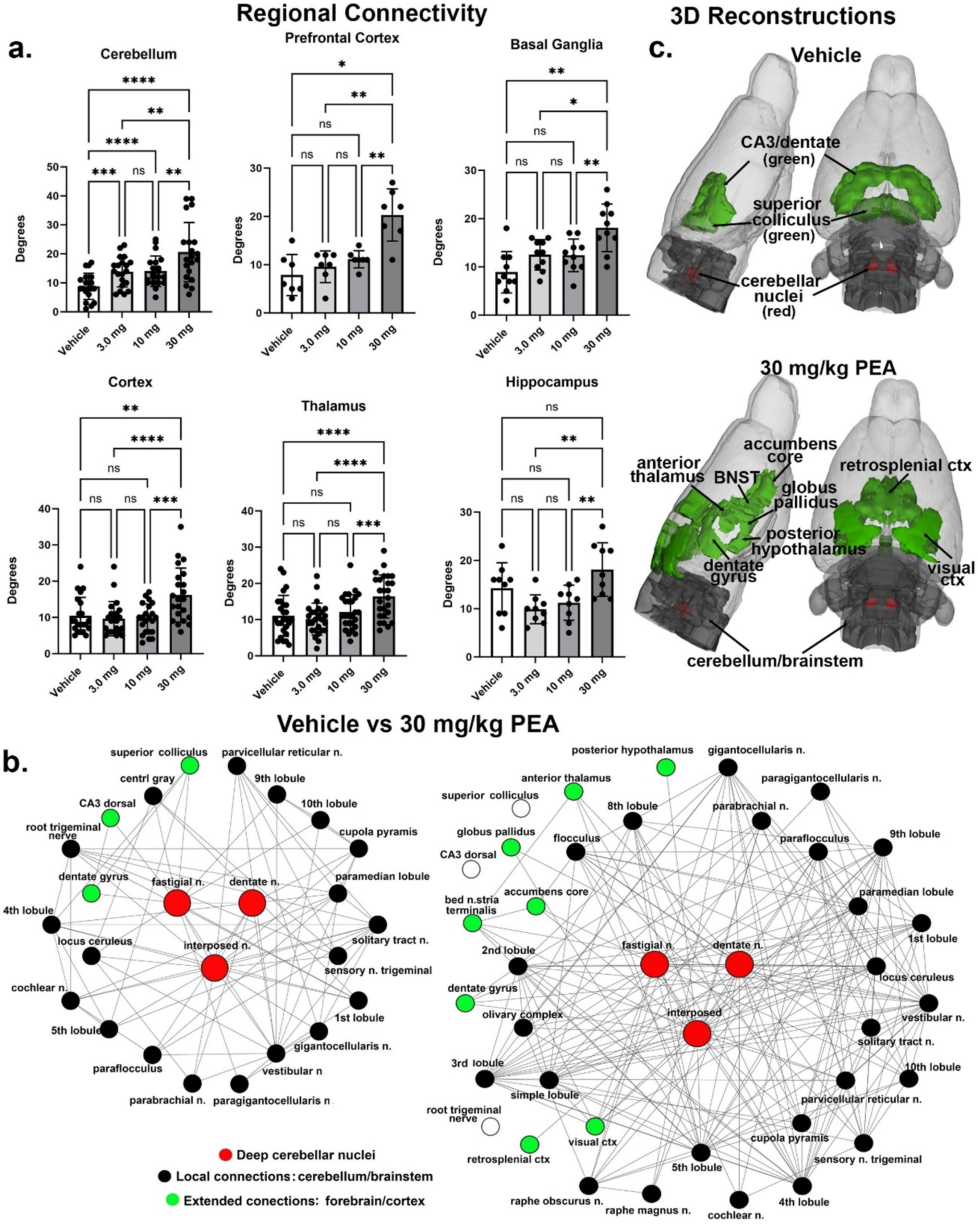
Functional connectivity. Shown in **(A)** are the number of degrees for the four main brain regions: primary somatosensory cortices, thalamus, prefrontal cortex, and basal ganglia. **(B)** Shows the radial representations of the connections or degrees (lines) to the three cerebellar nuclei (center red), e.g., fastigial, dentate and interposed following vehicle or 30 mg/kg PEA treatment. The black dots are brain areas within the cerebellum and immediate brainstem. Green dots are brain areas outside the hindbrain and more rostral. Note with PEA treatment the number of green dots increase as the deep cerebellar nuclei extent their connections to include visual cortex, accumbens and anterior thalamus as examples. **(C)** Is a 3D reconstruction of these brain areas colored coded black and green for vehicle and PEA treatments. All brain areas highlighted have direct connections to the cerebellar nuclei under each experimental condition. The significant difference in connectivity (Student *t*-test, ****p < 0.0001) is shown in the inserted bar graph at the center of the figure. *p < 0.05; **p < 0.01; ***p < 0.001.

Results from graph analysis looking at the number of connections (degrees) between the 173 different brain areas in the 3D MRI rat atlas for each PEA dose and vehicle are summarized by brain regions in Figure 4A. In all cases the 30 mg/kg dose increased brain connectivity. The dose-dependent nature of this effect is shown in the cerebellum where all doses are greater than vehicle and the 30 mg dose is greater than the 3 and 10 mg/kg doses [*F*_(1.728, 34.18)_ = 25.198, *p* < 0.0001]. The connectivity of the cerebellum to specific brain areas is shown in Figure 4B. All efferent information leaving the cerebellum essentially goes through the three deep cerebellar nuclei, e.g., dentate, fastigial and interposed highlighted in red. Following vehicle treatment their local connections are limited to surrounding cerebellar and brainstem areas (black circles), and a few extended connections, e.g., superior colliculus, CA3 and dentate gyrus of the hippocampus (green circles). Following the 30 mg/kg dose of PEA there is a significant increase in the number of degrees to local areas within the cerebellum and brainstem and to several more extended connections that include the accumbens, bed nucleus of the stria terminal (BNST), thalamus, hypothalamus, and visual cortex. Areas that were connected with vehicle treatment but not PEA treatment are shown as open white circles. These data are summarized in the 3D reconstructions in Figure 4C.

### Lipidomics outcomes with acute PEA

**Figure 5:**
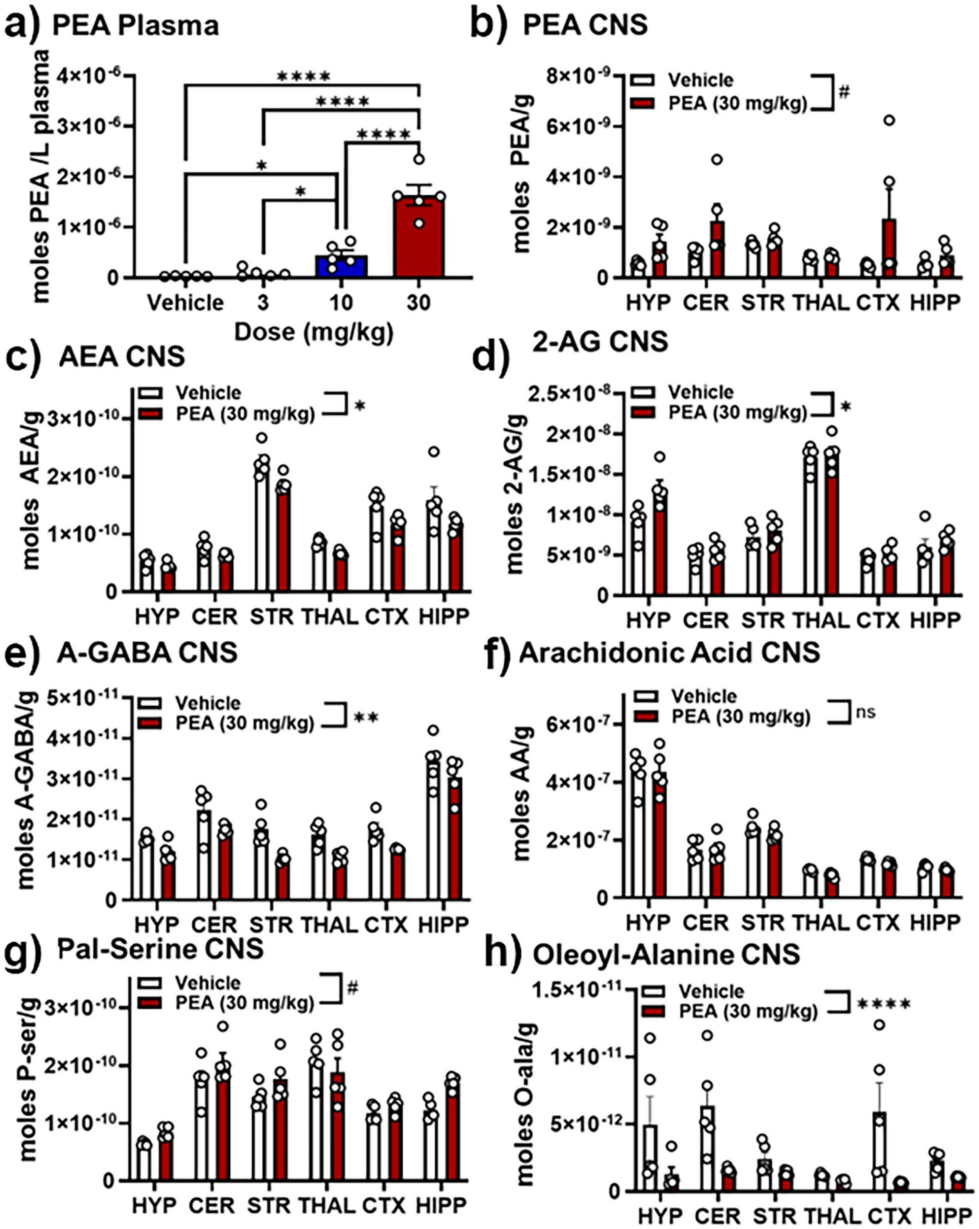
Effects of PEA injection on levels of select lipids in the plasma and CNS. **(A)** Levels of PEA in plasma 30 min after vehicle, 3, 10, and 30 mg/kg PEA *i.p.* injection. **(B)** PEA levels (moles/gram tissue) in 6 brain regions (hypothalamus, HYP; cerebellum, CER; striatum (caudate/putamen), STR; thalamus, THAL; anterior frontal cortex, CTX; hippocampus, HIPP) 30 min after vehicle (white bars) and 30 mg/kg PEA injection (red bars). **(C–H)** Show the levels of the following endogenous lipids in those same 6 brain regions 30 min after vehicle and 30 mg/kg PEA injection: **(C)** Anandamide (AEA), **(D)** 2-arachidonoyl glycerol (2-AG), **(E)** *N*-arachidonoyl Gamma-aminobutyric acid (A-GABA); **(F)** Arachidonic Acid (AA); **(G)** *N*-palmitoyl serine (Pal-Ser); and **(H)** *N*-oleoyl alanine. #*p* < 0.1–0.051; *p < 0.05; **p < 0.01, ***and ****p < 0.001, ns = not significant.

**Figure 6:**
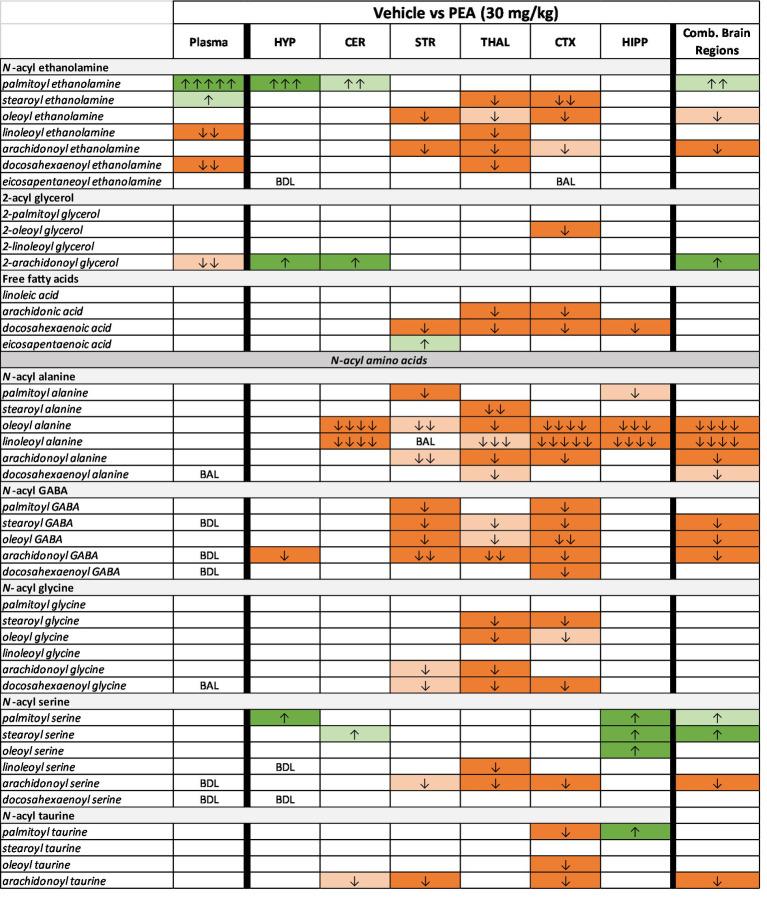
Heatmap of selected lipids in plasma and CNS after acute 30 mg/kg PEA injection. Analysis that supports the heatmap output is outlined in detail in the Methods. Data are shown as the levels of lipids in the 30 mg/kg PEA injected subjects compared to the vehicle control. Dark orange indicated significantly (*p* < 0.05) reduced levels of a specific lipid in the PEA group and light orange significance is *p* < 0.1–0.051. Likewise, dark and light green indicate significant increases of the specific lipid in the PEA group compared to the vehicle group. Arrows indicated fold-change. See Methods for key. BDL = below detectable limits. BAL = below analytical limits, which means that some samples had clearly discernible chromatographic peaks for the lipid and others did not and the overall levels were below the number needed for statistical Power to make a clear determination.

Figure 5, 6 illustrate changes in the plasma and CNS lipidome after acute PEA administration. Figure 6 is a selected heatmap (41 of 84) of the endogenous lipids analyzed in 6 brain regions and plasma after the 30 mg PEA/kg dose. See Supplementary Figure S5 for full heatmap. Figure 5 provides bar graphs with individual data points of selected data to illustrate the types of data used for generating the heatmap in Figure 6. Figure 5A shows the dose–response values of plasma PEA after 3, 10, and 30 mg/kg injections. These data show that PEA levels in the 30 mg/kg dose are 50-fold higher than those in the vehicle-injected group. This is illustrated in Figure 6 as five arrows denoting the largest fold increase in our scale (see Methods: MS analysis and Figure 3). Levels of PEA in hypothalamus (HYP) and cerebellum (CER) are also significantly increased when compared to vehicle control within brain region. When brain regions are combined, the overall levels of PEA are significantly higher than vehicle though the levels in the PEA-injection group show significantly more variability within group than the vehicle with some individual values that are more than 3-fold higher than vehicle (Figure 5B). Conversely, the levels of the endogenous cannabinoid, Anandamide (*N*-arachidonoyl ethanolamine; AEA) are significantly decreased in the striatum (STR), thalamus (THAL), and anterior cortex (CTX; Figure 6), which also translated in an overall decrease across the CNS (Figures 5C, 6). Likewise, in addition to individual brain regions (Figure 6), levels of *N*-oleoyl alanine (Ol-Ala; Figure 5H) were significantly reduced across the brain with some levels being more than 3-fold lower than the vehicle levels. Conversely, levels of 2-AG and *N*-palmitoyl serine (Pal-Ser; Figure 5G) were moderately but significantly increased across the brain with low variability in the levels in both groups, whereas levels of arachidonic acid (AA) that were significantly reduced in THAL and CTX did not demonstrate and overall reduction across the brain and exhibited low variability (Figure 5F).

**Figure 7:**
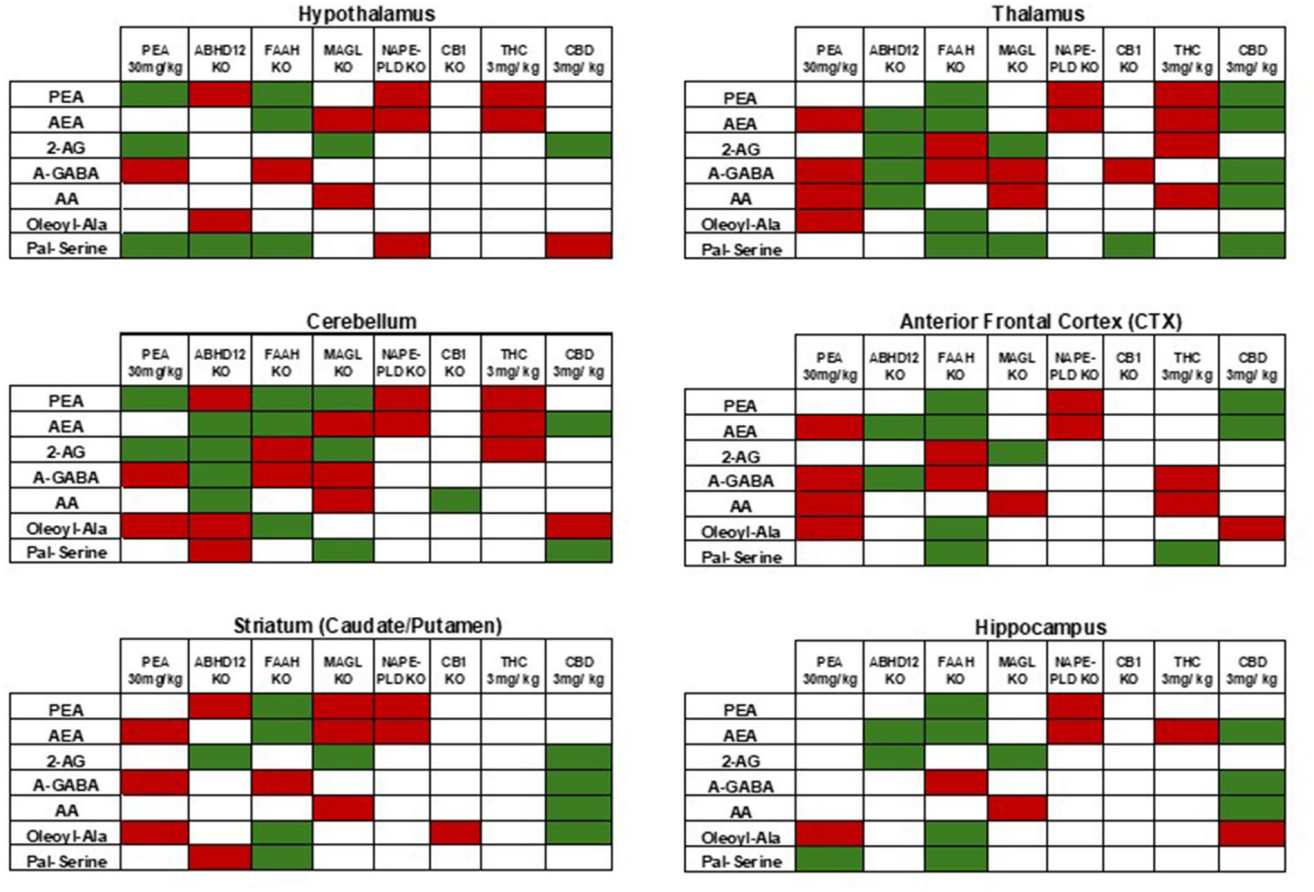
Comparisons of levels of selected CNS lipids in KO models and cannabinoid treatments to current PEA injection. As described in the *Discussion*, these comparisons are generated from previous reports (see references in text). Here, green indicates significant increases and red indicates significant decreases. In each case, the relative differences are either to baseline WT or vehicle depending on the study. Data from the current study of acute 30 mg PEA/kg is in the far-left column in each table. Tables are separated by CNS areas and the specific area is represented as a label at the top of each table.

Addition endolipids were modulated both in individual brain regions and across the brain (Figure 6; Supplementary material), with the most notable changes being that *N*-acyl alanine, *N*-acyl GABA, and *N*-acyl glycines all had multiple lipid species within those families that had significant decreases in the STR, THAL, and CTX. Supplementary Figure S3 likewise shows that additional lipids in the N-acyl tyrosine and N-acyl valine families were also reduced in STR, THAL, and CTX. For the purposes of discussion and insights into potential mechanisms of action, we will focus on the modulations of the 7 endolipids highlighted here (e.g., PEA, AEA, 2-AG, A-GABA, AA, Ol-Ala, and Pal-Ser; Figures 5, 7).

## Discussion

PEA is a popular, commercially available nutraceutical marketed as a dietary supplement but used for the treatment of numerous physical and mental maladies. Well controlled, clinical trials report efficacy in chronic musculoskeletal pain (Scuteri et al., 2022), diabetic neuropathy (Pickering et al., 2022), thermal pain in healthy volunteers (Lang-Illievich et al., 2022) and pain from acute respiratory infections (Masek et al., 1974). A recent meta-analysis using a sample of 774 patents from multiple studies provides evidence that PEA is effective in reducing chronic pain and enhancing quality of life (Lang-Illievich et al., 2023). When used in combination with traditional medicines it reduces symptoms of mania (Abedini et al., 2022), and psychosis (Salehi et al., 2022). Despite these many clinical reports of remediation of pain and symptoms of mental illness there is no evidence that PEA taken orally or systemically has an immediate effect on brain function and CNS lipid signaling. The present studies were designed to understand the effects of acute PEA on brain activity, behavior, and modulations of the circulating and CNS lipidome.

Myriad studies in animals and humans have given PEA orally in an ultra-micronized (PEA-um) formulation for improved bioavailability (Petrosino and Di Marzo, 2017; Impellizzeri et al., 2014), or in combination with luteolin (Lut), a vegetable flavonoid with anti-oxidant properties (Paterniti et al., 2014). Many of these studies (for reviews see Petrosino and Di Marzo, 2017; Colizzi et al., 2022) gave chronic oral doses of PEA to treat animals and patients with painful and inflammatory conditions. Few have looked at the immediate effects of PEA on behavior and biodistribution in healthy animals (Impellizzeri et al., 2014; Petrosino et al., 2018). Petrosino and coworkers gave an oral dose of 30 mg/kg [^13^C]_4_-PEA-um to healthy male SD rats and reported a rise in plasma levels from 1 to 22 pmoL/mL within 30 min of gavage (Petrosino et al., 2018). Whole brain-homogenate levels of [^13^C]_4_-PEA-um rose to 16 pmol/gm within 15 min of gavage. Carbon labeled PEA-um was used to distinguish between endogenous PEA. This study by Petrosino et al., clearly shows PEA gets into the brain at levels comparable to those in plasma and suggests a high brain volume of distribution.

BOLD imaging in awake rodents has been used to characterize the immediate effects of numerous drugs on brain activity (Ferris, 2022; Sadaka et al., 2021; Ferris et al., 2017; Ferris et al., 2015; Ghaw et al., 2024). In this case, the fingerprint or pattern of BOLD activity across 173 brain areas represents a complex interaction between different receptors across discrete brain areas. Pharmacological MRI is a method to assess the global integrated neural circuitry underpinning the behavioral effects of CNS therapeutics independent of their specific biochemical mechanism (Borsook et al., 2006). Two or more doses of drug plus vehicle are given systemically, e.g., i.p, s.c., or i.v., during the scanning session (Ferris, 2022). These studies are rarely done with oral dosing (Brevard et al., 2006) because of the invasive nature of a gastric tube or swallowing a liquid administered into the mouth. The solubility of the test compound dictates the vehicle. In this case, where PEA is highly lipophilic, it was necessary to use gum Arabic as a vehicle. Gum Arabic is a natural plant-derived substance commonly used in the pharmaceutical industry to emulsify lipophilic drugs for systemic administration.

Do the doses used in this study (3 mg/kg, 10 mg/kg, and 30 mg/kg) reflect the human experience? The standard dose of PEA for humans ranges from 300 mg to 600 mg taken twice daily (total daily dose of 600 mg to 1,200 mg). In a 70 kg person this would be approximately 8.6 mg/kg × 2. When considering allometric scaling, rodent doses are always higher as compared to humans given their higher metabolism and larger surface-to-volume ratio. The acute dose-dependent effects of PEA in this study were unexpected. The lowest dose had the greatest effect on increasing negative BOLD signal, a response that reflects a decrease in brain activity. Indeed for many brain areas there was an inverse negative BOLD dose response. This change in brain activity was primarily localized to the prefrontal cortex, basal ganglia, thalamus and sensorimotor cortices. The significance of these brain areas should not be underestimated as they form the cortico-basal ganglia-thalamic-cortical loop involved in sensory filtering and organization of sensory-motor stimuli (Banushi and Polito, 2023). This circuitry has been implicated in several psychiatric disorders.

There was little to no dose-dependent effect of PEA on global rsFC. The resulting hyperconnectivity was due primarily to the 30 mg/kg dose. The same cortico-basal ganglia-thalamic-cortical circuitry was impacted highlighting the sensitivity of these brain regions to PEA. In addition, the hippocampus and cerebellum showed hyperconnectivity to PEA treatment. Figure 4 highlights the cerebellum as a brain area that has been largely overlooked in both preclinical and clinical research. The cerebellum’s involvement was expected, considering numerous imaging studies in awake animals have shown changes in cerebellar activity following the administration of cannabinoids, psychostimulants, ketamine, opioids, and neuropeptides (Kawazoe et al., 2022; Sadaka et al., 2021; Demaree et al., 2021; Coleman et al., 2022; Moore et al., 2016) and most recently the hallucinogen LSD (Ghaw et al., 2024). Traditionally known for its role in motor coordination, the cerebellum is also involved in autonomic functions like heart rate, blood pressure, and respiration (Krohn et al., 2023; Cavdar et al., 2001; Haines et al., 1984; Snider et al., 1976). Furthermore, it plays a significant role in emotional and cognitive functions (Zhang et al., 2023; Schmahmann, 2019; Adamaszek et al., 2017), feeding (Low et al., 2021), and addiction (Miquel et al., 2016). The cerebellum’s unique connectivity is characterized by efferent pathways that pass through the fastigial, interposed, and dentate nuclei, which maintain extensive bidirectional connections with various brain regions such as the thalamus, hypothalamus, limbic cortex, amygdala, hippocampus, and brainstem (Kebschull et al., 2023). It also receives a substantial portion of its nerve connections from the vestibular complex, which transmits auditory information from the ear to the cortex. In this study, cerebellar connections were found to extend to the olfactory bulb, frontal association cortex, and hippocampus. This extensive network and the cerebellum’s role in various behavioral and sensory functions prompt the question: could the cerebellum contribute to hallucinogenic effects that resemble psychosis? Given its involvement in cognitive, emotional, and sensory processes, the cerebellum might play a role in the wide range of symptoms and cognitive impairments observed in schizophrenia (Andreasen and Pierson, 2008).

There are numerous rodent studies of different pathological models using various vehicles to solubilize PEA for i.p. or s.c administration in doses ranging from 10 to 30 mg/kg (for review see Rankin and Fowler, 2020). For example, in a mouse model of Alzheimer’s disease (AD) caused by the intraventricular injection of amyloid β 25–35, daily s.c. injections with 10–30 mg/kg doses of PEA prevent the behavioral deficits caused by the protein. However, in this AD model PEA is ineffective in PPAR-α null mice (D'Agostino et al., 2012). In a similar AD model using rats exposed to intrahippocampal amyloid β 1–42 there is an increase in reactive gliosis that is reduced with daily i.p injections of 10 mg/kg PEA (Scuderi et al., 2014). The beneficial results were mediated at least in part through PPAR-α. Mice given oral doses of PEA-um (10 mg/kg) for 60 days show a reduction in inflammation and motor deficits when challenged with the dopaminergic toxin 1-methyl-4-phenyl-1,2,3,6-tetrahydropyridine (MPTP) (Crupi et al., 2018).

In this study the acute effects of PEA on different measures of locomotion, motivation and cognition were unremarkable. The time spent in the center of the open field was not significantly different between treatments as all rats spent on average 5 s or less avoiding the open area. This could be taken as a sign of normal vigilance toward risk of predation. There was, however, a dose-dependent decrease in distance traveled. The behavior of rats treated with the 30 mg/kg dose was most interesting as they traveled less and spent the least amount of time along the side walls, but significantly more time in the corners which would be judged to be the safest area in the open field. Is this a sign of enhanced vigilance or increased anxiety? Mice made obese by a high fat diet and treated for seven wks with oral PEA-um (30 mg/kg) show increased time in the center area and distance traveled as to compared obese untreated mice (Lama et al., 2022). The tail flick assay in the hot bath for pain sensitivity was also unremarkable as acute PEA had no effect on time to withdraw the tail as compared to vehicle. This is inconsistent with the many studies showing pronounced analgesic effects in different rodent models of pain; however, the acute timing of the treatment and the models are different than some of the previous studies evaluating pain responses. For example, in the mouse model of chemotherapy-induced neuropathy caused by treatment with paclitaxel, PEA given IP reduces the pain associated with cold allodynia 60 min after treatment (Donvito et al., 2016). This effect could be blocked with a PPAR-α antagonist receptor. Interestingly, the paclitaxel-induced allodynia could be reduced by administering PEA through multiple routes, e.g., intrathecal, intraplantar and directly into the brain via intracerebroventricular injection, evidence that PEA can act on both the peripheral and central nervous system to achieve therapeutic efficacy. In addition, PEA’s analgesic activity extends to models of spinal cord (Genovese et al., 2008) and sciatic nerve injury (Costa et al., 2008), acute and chronic inflammation (D'Agostino et al., 2009; LoVerme et al., 2006; Gaspar et al., 2021), and diabetic neuropathy (Donvito et al., 2015). These are all models of preexisting pain. In this study PEA was ineffective in an acute test of thermal sensitivity in normal healthy rats. Because there is no effect using the tail flick assay is not evidence that PEA is ineffective as an analgesic.

While data support the hypothesis that PEA is an endogenous ligand for peroxisome proliferator-activated receptor alpha (PPAR-a), both its endogenous and pharmacological effects likely occur through interactions with other proteins (e.g., receptors, enzymes) and modulations of multiple lipid signaling pathways, especially given the timing of the effects here. *In vitro* and *in vivo* animal studies have identified several potential ways PEA can affect the brain. For example, one hypothesis for its ability to elevate levels of AEA in some models is by competing with FAAH the primary enzyme for degrading both NAEs (Rankin and Fowler, 2020). Here, data show that acute PEA treatment causes *reductions* in AEA across the CNS, therefore, the hypothesis that PEA acts to inhibit FAAH is not supported in this acute PEA injection model.

An alternative working hypothesis is that exogenous PEA is modulating more than one enzyme that affects the lipidome (Bradshaw and Leishman, 2017). Figure 7 compares lipidomics data from 7 lipid species that showed differences in multiple brain regions in the current study to those same lipids in (e.g., HYP, CER, STR, THAL, CTX, and HIPP) of FAAH KO (Leishman et al., 2016), MAGL KO (Leishman et al., 2016), ABHD12 KO (Leishman et al., 2019), NAPE-PLD KO (Leishman et al., 2016), CB1 KO (Leishman et al., 2016), acute 3 mg/kg THC treatment (Leishman et al., 2018), and acute 3 mg/kg CBD treatment (Leishman et al., 2018). As observed in these comparisons, acute PEA causes a lipid profile in HYP and CER that has similarities to ABHD12 KO, FAAH KO, MAGL KO, and CBD treatment but not NAPE-PLD KO, CB1 KO or THC treatment. Conversely, the STR lipid profile of these specific lipids after PEA injection share more traits with NAPE-PLD KO, CB1 KO, whereas THAL and CTX have more similarities to THC treatment. Finally, the PEA injection lipid profile here in HIPP had the least similarities overall to any of these KO examples but had the most in common with the CBD treatment. These examples provide a context to consider that levels of endogenous lipids are differentially modulated by changes in enzyme and receptor activity that is region-specific in the CNS. Further, generating an overall hypothesis that PEA is driving changes the CNS universally through the modulation of a single enzyme is unlikely. These data mirror the overall data from the neuroimaging analysis in that the changes in the CNS lipidome are both widespread and variable.

## Summary

These studies are the first of their kind to look at the acute dose-dependent effects of exogenous PEA on changes in functional brain activity and connectivity as well as changes in the broad-scale plasma and CNS lipidome. Because acute PEA had significant and dramatic effects on brain connectivity and the lipidome, this illustrates that it is imperative that the use of this compound as a nutraceutical be further studied. The potential use of PEA as a nutraceutical for a variety of symptoms has the potential to be an important addition to alternative therapeutics. However, as with all truly effective therapeutics it is essential to more fully understand mechanisms of action and potential adverse effects. Here, we show that acute PEA significantly changes CNS function and biochemistry. How these changes (connectivity and lipid regulation) occur are exciting and are novel findings that will not only allow us to understand how PEA affects CNS function, but to further understand CNS function overall. The standard dose of PEA for humans ranges from 300 mg to 600 mg taken twice daily (total daily dose of 600 mg to 1,200 mg). In a 70 kg person this would be about 8.6 mg/kg. When you consider allometric scaling, rodent doses are always higher given their higher metabolism and larger surface-to-volume ratio as compared to humans. Our dose is only given once. All things considered; we could have probably given more to better reflect the human experience.

## Data Availability

The original contributions presented in the study are included in the article/Supplementary material, further inquiries can be directed to the corresponding author.
