# Supplementary material for: Palmitoylethanolamide causes dose-dependent changes in brain function and the lipidome
Source: Front Neurosci. 2024 Nov 27;18:1506352. doi: 10.3389/fnins.2024.1506352 (PMC11631868; doi:10.3389/fnins.2024.1506352)
Supplement: Supplementary file 1 [file Data_Sheet_1.docx]

**Supplementary Data Figure 1 Motion artifact associated with awake imaging** Shown is the degree of motion recorded over the 20 min imaging protocol. The data are reported as the mean and standard error in micrometers for axis X, Y, and Z from all rats (n=19) used in the functional imaging studies.

**
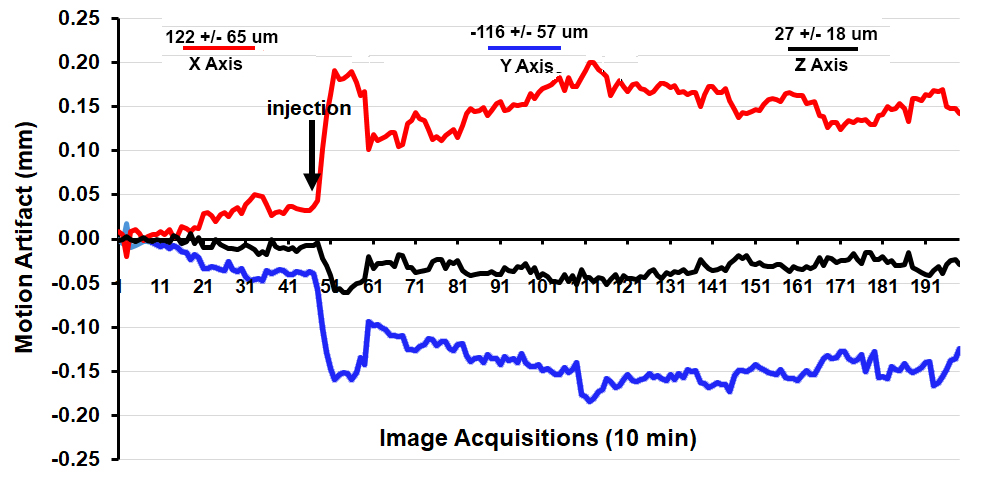
**

**Supplemental Figure 2 Identification of palmitoyl ethanolamine (PEA) in plasma of vehicle and PEA-treated rats**

PEA standards were scanned via MS/MS in positive mode for a parent mass of 300.3 and a fragment mass of 62.0 m/z, with a peak retention time of 5.01 minutes. Representative standard injections of 10 femtomoles (fmol) and 100 fmol are shown in A and B, respectively. Representative chromatograms of a 10 uL injection of methanolic plasma lipid extracts partially purified on C18 solid phase extraction (SPE) columns from rats treated with vehicle or 3, 10, or 30 mg/kg PEA (C-F, respectively). Unknown samples values were fit to a standard curve for final determination of PEA concentrations in plasma.

**Supplemental Figure 3 Representative chromatogram overlays of plasma and brain regions from vehicle or PEA-treated rats**

(A) Overlay chromatograms from plasma samples are shown for rats injected with vehicle (black line) or PEA (3 mg/kg, peach line) compared to a 10 fmol standard (dotted gray line). (B) Overlay chromatograms from plasma samples are shown for rats injected with or PEA at a dose of 10 (orange line) or 30 mg/kg (red line) compared to a 100 fmol standard (dashed gray line). (C) An overlay chromatogram from plasma samples from vehicle (black) and PEA (30 mg/kg, red) treated rats compared to a 100 fmol standard illustrates the magnitude with which PEA treatment elevated circulating PEA levels. (D) Representative chromatogram overlays of hypothalamus samples from vehicle (black) or PEA (30 mg/kg, red) treated rats compared to a 50 fmol standard. (E) Representative chromatogram overlays of cerebellum samples from vehicle (black) or PEA (30 mg/kg, red) treated rats compared to a 500 fmol standard.

**Supplementary Data Figure 4 Design of heatmap analysis.** Colors denote degrees of significant difference scores and arrows represent fold-changes from vehicle injection..

**Supplemental Figure 5 Full heatmap of significant differences in lipids measured from plasma and brain regions of vehicle and PEA-treated rats**

An unabbreviated heatmap of all quantified lipids in plasma and brain regions is given (A). Quantities of each analyte identified were compared in each tissue from vehicle and PEA (30 mg/kg) treated rats, using a two-tailed t-test in excel to determine statistical significance (A). Means of vehicle and PEA values were compared to evaluate fold change and direction of change for each analyte. Green boxes with an upward arrow indicate that analyte levels from PEA-treated rats were higher than vehicle-treated rats, whereas an orange box with downward arrow indicates lower levels of analyte in PEA-treated rat tissue compared to vehicle treated rats. Darker boxes represent comparisons that reached statistical significance (p< 0.05) while lighter shaded boxed represent a trending change in analyte level (p value between 0.1 and 0.051). Analyses in plasma and each brain region (hypothalamus, cerebellum, striatum, thalamus, cortex, hippocampus) are displayed independently to examine region specificity of analyte changes. Additionally, the sum total of analytes from all quantified brain regions (hypothalamus + cerebellum + striatum + thalamus + cortex + hippocampus) were compared between vehicle-treated rats and PEA-treated rats to generate a “Combined Brain Region” set of comparisons (A, far right column). Number of arrows displays the magnitude of change (see B for full legend).
